# Crystal structure and Hirshfeld surface analysis of a new di­thio­glycoluril: 1,4-bis­(4-meth­oxy­phen­yl)-3*a*-methyl­tetra­hydro­imidazo[4,5-*d*]imidazole-2,5(1*H*,3*H*)-di­thione

**DOI:** 10.1107/S2056989019010764

**Published:** 2019-08-06

**Authors:** Jonnie N. Asegbeloyin, Kenechukwu J. Ifeanyieze, Obinna C. Okpareke, Ebube E. Oyeka, Tatiana V. Groutso

**Affiliations:** a Department of Pure and Industrial Chemistry, University of Nigeria, Nsukka 410001, Enugu State, Nigeria; b School of Chemical Sciences, the University of Auckland Private Bag 92019, Auckland 1142, New Zealand

**Keywords:** crystal structure, thio­glycoluril, imidazole, cinnamoyl chloride, iso­thio­cyanate, N—H⋯S hydrogen bonding, C—H⋯π inter­actions

## Abstract

In the title di­thio­glycoluril derivative, C_19_H_20_N_4_O_3_S_2_, there is a difference in the torsion angles between the thio­imidazole moiety and the meth­oxy­phenyl groups on either side of the mol­ecule [C—N—C_ar_—C_ar_ = 116.9 (2) and −86.1 (3)°, respectively]. The N—C—N bond angle on one side of the di­thio­glycoluril moiety is slightly smaller compared to that on the opposite side, [110.9 (2)° *cf*. 112.0 (2)°], probably as a result of the steric effect of the methyl group. In the crystal, N—H⋯S hydrogen bonds link adjacent mol­ecules to form chains propagating along the *c-*axis direction. The chains are linked by C—H⋯S hydrogen bonds, forming layers parallel to the *bc* plane. The layers are then linked by C—H⋯π inter­actions, leading to the formation of a three-dimensional supra­molecular network. Hirshfeld surface analysis and two-dimensional fingerprint plots were used to investigate the mol­ecular inter­actions in the crystal.

## Abstract 2

In the title di­thio­glycoluril derivative, there is a difference in the torsion angles between the thio­imidazole moiety and the meth­oxy­phenyl groups on either side of the mol­ecule [C—N—C_ar_—C_ar_ = 116.9 (2) and −86.1 (3)°]. The N—C—N bond angle on one side of the di­thio­glycoluril moiety is slightly smaller than the one on the opposite side [110.9 (2)° *cf*. 112.0 (2)°], probably as a result of the steric effect of the methyl group.

## Chemical context   

Heterocycles with five-membered rings containing two nitro­gen atoms in the 1,3 positions and three carbon atoms in the ring are known as imidazoles. Most imidazoles, except for the N-substituted derivatives, have a distinct pyrrole type and pyridine-type annular nitro­gen atoms. The isolation of imidazole derivatives has been documented (Beyer *et al.*, 2011 ▸; Zeng *et al.*, 2003 ▸; Dawood *et al.*, 2010 ▸). Glycolurils, tetra­hydro­imidazo[4,5-*d*]imidazole-2,5(1*H*,3*H*)-diones, are well-known imidazole derivatives of great research inter­est. As well as serving as building blocks in the preparation of many organic compounds and supra­molecular synthons (Burnett *et al.*, 2003 ▸; Kravchenko *et al.*, 2018 ▸), they have also been reported to behave as nootropic (Ryzhkina *et al.*, 2013 ▸), neurotropic (Berlyand *et al.*, 2013 ▸) and anxiolytic agents (Kravchenko *et al.*, 2018 ▸)*.* Some derivatives are used as flame-resistant materials (Sal’keeva *et al.*, 2016 ▸; Zharkov *et al.*, 2015 ▸) and gelators (Tiefenbacher *et al.*, 2011 ▸). While several glycoluril analogues have been synthesized and characterized, reports on di­thio­glycolurils are quite rare. In the course of our search for thio­ureas with bioactivity, we had intended to isolate (2*E*)-*N*-[(4-meth­oxy­phen­yl)carbamo­thio­yl]-3-phenyl­prop-2-enamide using well-documented methods (Asegbeloyin *et al.*, 2018 ▸; Douglass & Dains, 1934 ▸; Oyeka *et al.*, 2018 ▸); however, we obtained crystals of 1,4-bis­(4-meth­oxy­phen­yl)-3*a*-methyl­tetra­hydro­imidazo[4,5-*d*]imidazole-2,5(1*H*,3*H*)-di­thione, a new di­thio­glycoluril. As a result of the importance of glucolurils and their analogues and our current inter­est in the construction of novel heterocycles with good bioactivity (Asegbeloyin *et al.*, 2019 ▸), we decided to investigate the title compound, and we report herein on its synthesis, crystal structure and Hirshfeld surface analysis.
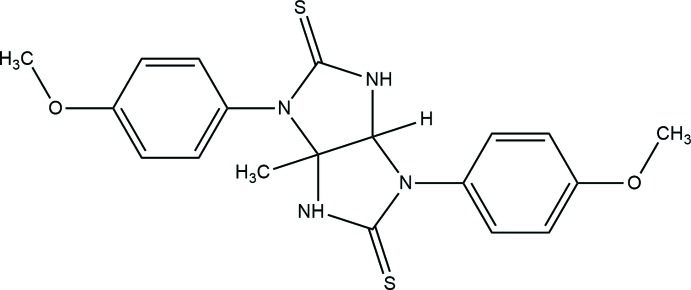


## Structural commentary   

**Figure 1:**
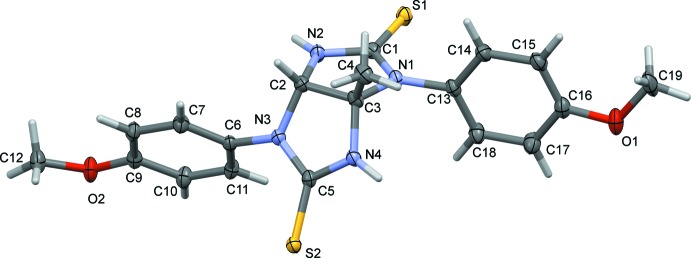
A view of the mol­ecular structure of the title compound, with the atom labelling. Displacement ellipsoids are drawn at the 30% probability level.

The mol­ecular structure and conformation of the title compound is shown in Fig. 1 ▸. The two imidazole rings, N1/N2/C1–C3 and N3/N4/C2/C3/C5, are inclined to each other by 62.16 (12)°, while the 4-meth­oxy­phenyl rings (C6–C11 and C13–C18) are inclined to each other by 29.36 (12)°. The latter rings are inclined to the imidazole ring to which they are attached by 62.51 (11) and 89.16 (12)°, respectively. Hence, the two ends of the mol­ecule are orientated differently, as shown by the difference in the torsion angles between the thio­imidazole moiety and the meth­oxy­phenyl groups; C2—N3—C6—C11 and C3—N1—C13—C14 are 116.9 (2) and −86.1 (3)°, respectively.

The thione C=S bond lengths of 1.674 (2) Å are longer than those in previous reports where all N atoms were substituted (Deng *et al.*, 2010 ▸; Wang *et al.*, 2011 ▸; Wu & Sun, 2009 ▸; Zhang *et al.*, 2011 ▸). The C—N bonds around the thione moiety [C1—N1, C1—N2, C5—N3 and C5—N4 = 1.350 (3), 1.357 (3), 1.355 (3) and 1.353 (3) Å, respectively] are significantly shorter than the average C—N single bond length of 1.48 Å (Oyeka *et al.*, 2018 ▸), as has also been observed in other thio­glycoluril systems (Wu & Sun, 2009 ▸; Zhang *et al.*, 2011 ▸) and acyl thio­urea derivatives (Asegbeloyin *et al.*, 2018 ▸; Oyeka *et al.*, 2018 ▸). This is probably due to the conjugation between the π-electrons on C=S and the lone pairs of electrons on the nitro­gen atoms. The C—C bond lengths of the aromatic rings are typical of *sp* ^2^-hybridized carbons while the C2—C3 bond of the thio­glycoluril moiety [1.542 (3) Å] shows *sp* ^3^ hybridization. These bond lengths are consistent with previous reports for thio­glycourils and acyl­thio­ureas (Binzet *et al.*, 2009 ▸; Oyeka *et al.*, 2018 ▸; Wang & Xi, 2009 ▸; Yang, 2010 ▸). The imidazole carbon atoms, C2 and C3, each have a distorted tetra­hedral geometry with the N1—C3—N4 and N2—C2—N3 bond angles being 112.0 (4) and 112.9 (2)°, respectively. The bond angles between the N-meth­oxy­phenyl nitro­gen atom and the aromatic ring, C5—N3—C6 and C1—N1—C13, are 124.8 (2) and 126.1 (2)°, respectively.

## Supra­molecular features   

**Figure 2:**
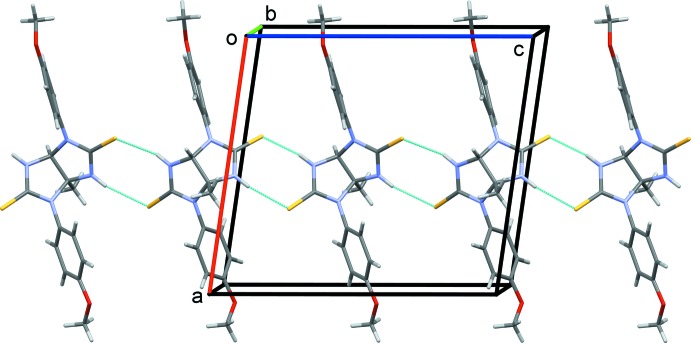
A view along the *b* axis of the N—H⋯S hydrogen-bonded chain in the crystal of the title compound.

**Figure 3:**
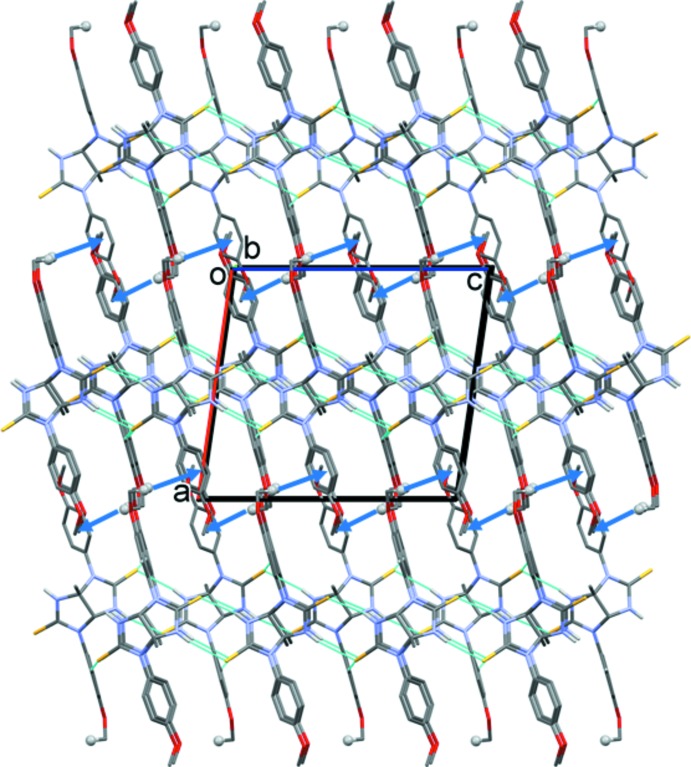
A view along the *b* axis of the crystal packing of the title compound. The hydrogen bonds are shown as dashed lines and the C—H⋯π inter­actions are represented by blue arrows (see Table 1 ▸ for details). For clarity, H atoms not involved in these inter­actions have been omitted.

In the crystal, N—H⋯S hydrogen bonds link neighbouring mol­ecules to form chains propagating along the *c*-axis direction (Table 1 ▸ and Fig. 2 ▸). The chains are linked by C—H⋯S hydrogen bonds, forming layers parallel to the *bc* plane (Fig. 3 ▸ and Table 1 ▸). In turn, the layers are linked by C—H⋯π inter­actions involving a meth­oxy methyl H atom (H12*B*) and a 4-meth­oxy­phenyl ring (C13–C18); see Table 1 ▸. These inter­actions result in the formation of a supra­molecular three-dimensional architecture (Fig. 3 ▸).

### Hirshfeld Surface Analysis   

**Figure 4:**
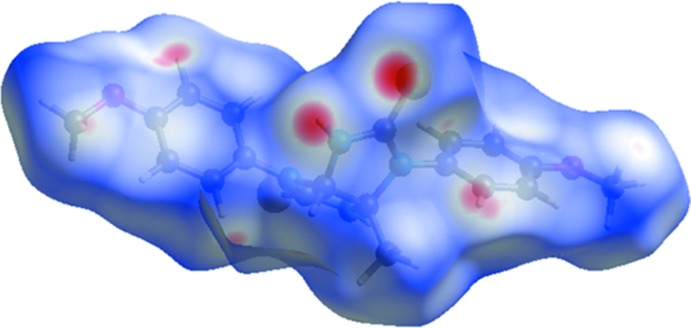
The Hirshfeld surface of the title compound mapped over *d*_norm_, with an arbitrary colour scale of −0.3207 to 1.4281.

**Figure 5:**
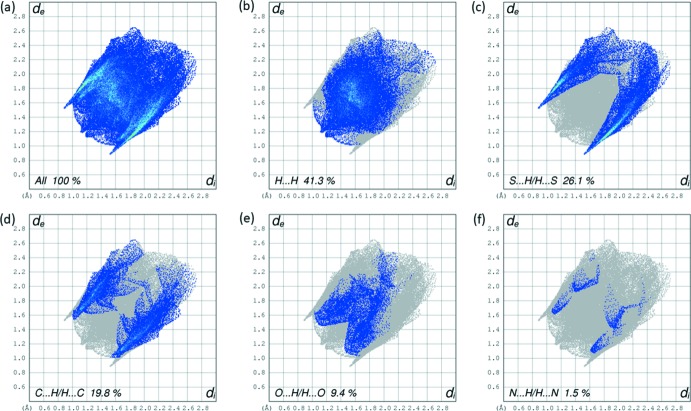
(*a*) The full two-dimensional fingerprint plot of the title compound, and fingerprint plots delineated into (*b*) H⋯H, (*c*) S⋯H/H⋯S, (*d*) C⋯H/H⋯C, (*e*) O⋯H/H⋯O and (*f*) N⋯H/H⋯N contacts.

The Hirshfeld surface analysis (Spackman & Jayatilaka, 2009 ▸) and the associated two-dimensional fingerprint plots (McKinnon *et al.*, 2007 ▸) were performed with *CrystalExplorer17* (Turner *et al.*, 2017 ▸). In the Hirshfeld surface mapped over *d*_norm_ (Fig. 4 ▸), the red spots indicate contacts shorter than the sum of the van der Waals radii with negative *d*_norm_, blue regions represent contacts longer than the sum of van der Waals radii with negative *d*_norm_, while white regions correspond to inter­molecular distances close to the sum of the van der Waals radii with *d*_norm_ equal to zero. The most intense red spots on the surface of the title compound are found around the thione S and N—H groups of the compound, which play a role in the hydrogen bonding inter­actions in the crystal (Table 1 ▸ and Fig. 2 ▸). The less intense red spots (Fig. 4 ▸), are observed around the ring carbon atoms resulting from C—H⋯S and C—H⋯π short contacts. The two-dimensional fingerprint plots (Fig. 5 ▸) show the overall contribution of the various inter­actions and those delineated into H⋯H, S⋯H/H⋯S, C⋯H/H⋯C, O⋯H/H⋯O and N⋯H/H⋯N contacts. Apart from the non-directional H⋯H contacts (41.3%), the highest contribution to the Hirshfeld surface is from S⋯H/H⋯S contacts (26.1%).

## Database survey   

A search of the Cambridge Structural Database (CSD, Version 5.39, February 2019) for thio­glycoluril found two mol­ecules similar to the title compound: 1,6-dipivaloyl-3,3a,4,6a-tetra­methyl­tetra­hydro­imidazo[4,5-*d*]imidazole-2,5(1*H*,3*H*)-di­thione (refcode ADEMOL; Duspara *et al.*, 2001 ▸) and 1,6-diacetyl-3,4,7,8-tetra­methyl-2,5-di­thio­gylcoluril (SOLQIT; Cow, 1998 ▸). In both compounds, large polar groups are substituted on adjacent sides of the imidazole ring, resulting in steric hindrance and distortion of the C—N—C angles. The C—N—C bond angles between the thione carbon and the N-substituted groups are *ca* 119.8 and 125.4° in ADEMOL and 122.6 and 125.4° in SOLQIT. In the title compound, the C5—N3—C6 and C1—N1—C13 bond angles are 124.8 (2) and 126.1 (2)°, respectively, showing only little distortion. The thione bond lengths [C5=S2 and C1=S1 are both 1.674 (2) Å] in the title compound are longer than in the reference compounds (1.650–1.664 Å). This is probably due to the fact that all of the imidazole nitro­gen atoms in the reference compounds are substituted. The presence of unsubstituted imidazole nitrogens in the title compound promotes conjugation between the lone pairs of electrons on the nitro­gen atom and the C=S π-electrons and hence stretches the C=S bond. The C—N bond lengths around the thione group of the title compound [1.350 (3)–1.357 (3) Å] are shorter than the corresponding bonds in the reference compounds (*ca* 1.367–1.397 Å). The other C—N bonds and the C—C bonds in the thio­gylcouril moiety are similar to those of the title compound.

## Synthesis and crystallization   

The title compound was synthesized according to the reported method (Asegbeloyin *et al.*, 2018 ▸; Douglass & Dains, 1934 ▸; Oyeka *et al.*, 2018 ▸). A solution of cinnamoyl chloride (0.02 mol) dissolved in 40 ml acetone was mixed with a 30 ml acetone solution of potassium thio­cyanate (0.02 mol). The reaction mixture was refluxed for 30 min to give a suspension of cinnamoyl iso­thio­cyanate, which was then left to cool to room temperature. 4-Meth­oxy­aniline (0.02 mol) was dissolved in 40 ml of acetone and the resulting solution was mixed with the suspension of cinnamoyl iso­thio­cyanate, and the mixture was stirred for 2 h. The resultant lemon–green solution was filtered and left at room temperature for 96 h to obtain colourless plate-like crystals of the title compound.

## Refinement   

Crystal data, data collection and structure refinement details are summarized in Table 2 ▸. Hydrogen atoms were placed at idealized positions (N—H = 0.86 Å, C—H = 0.93–0.98 Å) and refined using a riding model with *U*_iso_(H) = 1.5*U*_eq_(C-meth­yl) and 1.2*U*_eq_(C,N) for other H atoms.

## Supplementary Material

Crystal structure: contains datablock(s) I. DOI: 10.1107/S2056989019010764/ex2021sup1.cif

Structure factors: contains datablock(s) I. DOI: 10.1107/S2056989019010764/ex2021Isup2.hkl

Supporting information file. DOI: 10.1107/S2056989019010764/ex2021Isup4.cml

supporting information. DOI: 10.1107/S2056989019010764/ex2021sup3.docx

CCDC reference: 1906113

Additional supporting information:  crystallographic information; 3D view; checkCIF report

## Figures and Tables

**Table 1:** Hydrogen-bond geometry (Å, °) *Cg* is the centroid of the C13–C18 ring.

*D*—H⋯*A*	*D*—H	H⋯*A*	*D*⋯*A*	*D*—H⋯*A*
N2—H2⋯S2^i^	0.86	2.62	3.265 (2)	133
N4—H4⋯S1^ii^	0.86	2.57	3.382 (2)	157
C7—H7⋯S1^iii^	0.93	2.87	3.786 (3)	170
C12—H12*B*⋯*Cg* ^iv^	0.96	2.99	3.893 (3)	157

Symmetry codes: (i) 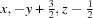; (ii) 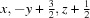; (iii) 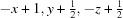; (iv) 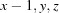.

**Table 2:** Experimental details

Crystal data	
Chemical formula	C_19_H_20_N_4_O_2_S_2_
*M*_r_	400.51
Crystal system, space group	Monoclinic, *P*2_1_/*c*
Temperature (K)	100
*a*, *b*, *c* (Å)	13.1955 (3), 10.0157 (2), 14.5476 (3)
β (°)	98.329 (2)
*V* (Å^3^)	1902.36 (7)
*Z*	4
Radiation type	Cu *K*α
μ (mm^−1^)	2.73
Crystal size (mm)	0.12 × 0.05 × 0.01

Data collection	
Diffractometer	Rigaku Oxford Diffraction XtaLAB Synergy, Dualflex, Pilatus 200K
Absorption correction	Multi-scan (*CrysAlis PRO*; Rigaku OD, 2018 ▸)
*T*_min_, *T*_max_	0.735, 1.000
No. of measured, independent and observed [*I* > 2σ(*I*)] reflections	25390, 3824, 3267
*R*_int_	0.053
(sin θ/λ)_max_ (Å^−1^)	0.624

Refinement	
*R*[*F* ^2^ > 2σ(*F* ^2^)], *wR*(*F* ^2^), *S*	0.047, 0.156, 1.22
No. of reflections	3824
No. of parameters	247
H-atom treatment	H-atom parameters constrained
Δρ_max_, Δρ_min_ (e Å^−3^)	0.63, −0.53

Computer programs: *CrysAlis PRO* (Rigaku OD, 2018 ▸), *olex2.solve* (Bourhis *et al.*, 2015 ▸), *SHELXL2016/6* (Sheldrick, 2015 ▸), *Mercury* (Macrae *et al.*, 2008 ▸) and *OLEX2* (Dolomanov *et al.*, 2009 ▸).

## supplementary crystallographic information

### Crystal data

**Table:**

C_19_H_20_N_4_O_2_S_2_	*F*(000) = 840
*M**_r_* = 400.51	*D*_x_ = 1.398 Mg m^−^^3^
Monoclinic, *P*2_1_/*c*	Cu *K*α radiation, λ = 1.54184 Å
*a* = 13.1955 (3) Å	Cell parameters from 11825 reflections
*b* = 10.0157 (2) Å	θ = 3.4–73.8°
*c* = 14.5476 (3) Å	µ = 2.73 mm^−^^1^
β = 98.329 (2)°	*T* = 100 K
*V* = 1902.36 (7) Å^3^	Plate, clear colourless
*Z* = 4	0.12 × 0.05 × 0.01 mm

### Data collection

**Table:**

Rigaku Oxford Diffraction XtaLAB Synergy, Dualflex, Pilatus 200K diffractometer	3824 independent reflections
Radiation source: micro-focus sealed X-ray tube, PhotonJet (Cu) X-ray Source	3267 reflections with *I* > 2σ(*I*)
Mirror monochromator	*R*_int_ = 0.053
ω scans	θ_max_ = 74.2°, θ_min_ = 3.4°
Absorption correction: multi-scan (CrysAlisPro; Rigaku OD, 2018)	*h* = −16→15
*T*_min_ = 0.735, *T*_max_ = 1.000	*k* = −12→12
25390 measured reflections	*l* = −18→15

### Refinement

**Table:**

Refinement on *F*^2^	Primary atom site location: iterative
Least-squares matrix: full	Hydrogen site location: inferred from neighbouring sites
*R*[*F*^2^ > 2σ(*F*^2^)] = 0.047	H-atom parameters constrained
*wR*(*F*^2^) = 0.156	*w* = 1/[σ^2^(*F*_o_^2^) + (0.1*P*)^2^] where *P* = (*F*_o_^2^ + 2*F*_c_^2^)/3
*S* = 1.22	(Δ/σ)_max_ = 0.001
3824 reflections	Δρ_max_ = 0.63 e Å^−^^3^
247 parameters	Δρ_min_ = −0.53 e Å^−^^3^
0 restraints	

### Special details

**Table:**

Geometry. All esds (except the esd in the dihedral angle between two l.s. planes) are estimated using the full covariance matrix. The cell esds are taken into account individually in the estimation of esds in distances, angles and torsion angles; correlations between esds in cell parameters are only used when they are defined by crystal symmetry. An approximate (isotropic) treatment of cell esds is used for estimating esds involving l.s. planes.

### Fractional atomic coordinates and isotropic or equivalent isotropic displacement parameters (Å^2^)

**Table:**

	*x*	*y*	*z*	*U*_iso_*/*U*_eq_	
S2	0.42015 (4)	0.63758 (5)	0.56357 (4)	0.02883 (19)	
S1	0.69303 (4)	0.64039 (6)	0.20550 (4)	0.03272 (19)	
O2	0.05235 (12)	0.52239 (19)	0.24656 (13)	0.0411 (4)	
O1	1.06251 (13)	0.6869 (2)	0.55605 (14)	0.0501 (5)	
N3	0.42969 (13)	0.73812 (19)	0.39262 (13)	0.0282 (4)	
N1	0.66847 (13)	0.76111 (19)	0.36715 (13)	0.0282 (4)	
N4	0.56744 (13)	0.7746 (2)	0.49428 (13)	0.0302 (4)	
H4	0.607926	0.773181	0.546222	0.036*	
N2	0.52813 (13)	0.7489 (2)	0.26451 (13)	0.0321 (4)	
H2	0.488712	0.728354	0.214118	0.039*	
C6	0.33311 (15)	0.6860 (2)	0.35217 (14)	0.0278 (4)	
C5	0.47370 (15)	0.7169 (2)	0.48150 (15)	0.0269 (4)	
C13	0.77144 (15)	0.7412 (2)	0.41232 (15)	0.0285 (5)	
C2	0.49454 (16)	0.8198 (2)	0.34048 (15)	0.0288 (5)	
H2A	0.462130	0.905134	0.320659	0.035*	
C9	0.14274 (16)	0.5846 (2)	0.27955 (16)	0.0326 (5)	
C1	0.62924 (16)	0.7179 (2)	0.28148 (15)	0.0290 (4)	
C3	0.59244 (16)	0.8391 (2)	0.41109 (16)	0.0294 (5)	
C11	0.31898 (16)	0.5490 (2)	0.34585 (16)	0.0326 (5)	
H11	0.373181	0.491552	0.365398	0.039*	
C10	0.22374 (17)	0.4982 (2)	0.31029 (17)	0.0355 (5)	
H10	0.213660	0.406289	0.306848	0.043*	
C8	0.15769 (17)	0.7205 (3)	0.28276 (17)	0.0346 (5)	
H8	0.104211	0.778010	0.261024	0.041*	
C7	0.25360 (17)	0.7716 (3)	0.31882 (17)	0.0345 (5)	
H7	0.264271	0.863400	0.320503	0.041*	
C14	0.84562 (18)	0.8354 (3)	0.40334 (17)	0.0370 (5)	
H14	0.829035	0.910173	0.366309	0.044*	
C16	0.96858 (17)	0.7111 (3)	0.50581 (18)	0.0383 (6)	
C15	0.94482 (18)	0.8197 (3)	0.44908 (17)	0.0393 (6)	
H15	0.994904	0.882321	0.441325	0.047*	
C18	0.79544 (19)	0.6308 (2)	0.4673 (2)	0.0383 (6)	
H18	0.745801	0.566657	0.473044	0.046*	
C4	0.62578 (19)	0.9834 (3)	0.42760 (19)	0.0391 (6)	
H4A	0.685302	0.986734	0.474091	0.059*	
H4B	0.571319	1.033392	0.448355	0.059*	
H4C	0.641782	1.021121	0.370769	0.059*	
C12	−0.03793 (18)	0.6032 (3)	0.2306 (2)	0.0427 (6)	
H12A	−0.033051	0.663519	0.180189	0.064*	
H12B	−0.044452	0.653243	0.285733	0.064*	
H12C	−0.096868	0.546930	0.215268	0.064*	
C17	0.8940 (2)	0.6156 (3)	0.5143 (2)	0.0432 (6)	
H17	0.910343	0.541071	0.551726	0.052*	
C19	1.14391 (19)	0.7756 (4)	0.5431 (2)	0.0588 (9)	
H19A	1.151078	0.778830	0.478399	0.088*	
H19B	1.206604	0.744313	0.578239	0.088*	
H19C	1.128777	0.863354	0.563931	0.088*	

### Atomic displacement parameters (Å^2^)

**Table:**

	*U*^11^	*U*^22^	*U*^33^	*U*^12^	*U*^13^	*U*^23^
S2	0.0231 (3)	0.0352 (3)	0.0281 (3)	−0.00311 (18)	0.0035 (2)	0.00066 (19)
S1	0.0274 (3)	0.0385 (3)	0.0323 (3)	0.0050 (2)	0.0046 (2)	−0.0049 (2)
O2	0.0238 (8)	0.0425 (10)	0.0540 (11)	0.0022 (7)	−0.0052 (7)	−0.0128 (8)
O1	0.0242 (8)	0.0612 (13)	0.0617 (12)	0.0036 (8)	−0.0043 (8)	−0.0093 (10)
N3	0.0207 (8)	0.0359 (10)	0.0283 (9)	−0.0014 (7)	0.0043 (7)	0.0021 (7)
N1	0.0203 (8)	0.0334 (10)	0.0313 (9)	0.0005 (7)	0.0049 (7)	−0.0038 (7)
N4	0.0218 (8)	0.0410 (11)	0.0281 (9)	−0.0041 (7)	0.0050 (7)	−0.0020 (8)
N2	0.0223 (8)	0.0439 (11)	0.0298 (10)	0.0034 (8)	0.0028 (7)	−0.0014 (8)
C6	0.0218 (10)	0.0368 (12)	0.0245 (10)	0.0012 (8)	0.0031 (8)	−0.0011 (9)
C5	0.0219 (9)	0.0290 (10)	0.0299 (10)	0.0014 (8)	0.0037 (8)	−0.0034 (8)
C13	0.0230 (10)	0.0336 (11)	0.0294 (11)	−0.0037 (8)	0.0055 (8)	−0.0073 (9)
C2	0.0253 (10)	0.0321 (11)	0.0298 (11)	0.0019 (8)	0.0069 (8)	0.0021 (9)
C9	0.0251 (11)	0.0397 (12)	0.0319 (11)	0.0037 (9)	0.0006 (8)	−0.0074 (9)
C1	0.0279 (10)	0.0298 (10)	0.0295 (11)	−0.0001 (8)	0.0055 (8)	0.0012 (8)
C3	0.0261 (10)	0.0322 (11)	0.0318 (11)	−0.0005 (8)	0.0101 (8)	−0.0035 (9)
C11	0.0248 (10)	0.0362 (12)	0.0349 (12)	0.0074 (9)	−0.0020 (8)	−0.0034 (9)
C10	0.0291 (11)	0.0322 (12)	0.0430 (13)	0.0016 (9)	−0.0027 (9)	−0.0046 (10)
C8	0.0251 (10)	0.0397 (13)	0.0378 (12)	0.0081 (9)	0.0012 (9)	0.0006 (10)
C7	0.0286 (11)	0.0341 (12)	0.0397 (12)	0.0022 (9)	0.0010 (9)	0.0007 (10)
C14	0.0289 (11)	0.0451 (13)	0.0368 (12)	−0.0083 (10)	0.0041 (9)	0.0045 (10)
C16	0.0259 (11)	0.0487 (14)	0.0395 (13)	0.0019 (10)	0.0018 (9)	−0.0091 (11)
C15	0.0282 (11)	0.0519 (15)	0.0387 (13)	−0.0098 (10)	0.0074 (9)	−0.0026 (11)
C18	0.0281 (11)	0.0340 (12)	0.0517 (15)	−0.0039 (9)	0.0020 (10)	−0.0011 (10)
C4	0.0382 (12)	0.0334 (12)	0.0478 (14)	−0.0015 (10)	0.0128 (10)	−0.0048 (10)
C12	0.0240 (11)	0.0507 (15)	0.0518 (15)	0.0035 (10)	0.0002 (10)	−0.0070 (12)
C17	0.0343 (13)	0.0375 (13)	0.0557 (16)	0.0026 (10)	−0.0005 (11)	0.0040 (11)
C19	0.0237 (12)	0.103 (3)	0.0501 (16)	−0.0083 (14)	0.0054 (11)	−0.0106 (16)

### Geometric parameters (Å, º)

**Table:**

S2—C5	1.674 (2)	C9—C8	1.375 (4)
S1—C1	1.674 (2)	C3—C4	1.520 (3)
O2—C9	1.370 (3)	C11—H11	0.9300
O2—C12	1.431 (3)	C11—C10	1.385 (3)
O1—C16	1.366 (3)	C10—H10	0.9300
O1—C19	1.427 (4)	C8—H8	0.9300
N3—C6	1.423 (3)	C8—C7	1.395 (3)
N3—C5	1.355 (3)	C7—H7	0.9300
N3—C2	1.472 (3)	C14—H14	0.9300
N1—C13	1.435 (3)	C14—C15	1.389 (3)
N1—C1	1.350 (3)	C16—C15	1.374 (4)
N1—C3	1.487 (3)	C16—C17	1.391 (4)
N4—H4	0.8600	C15—H15	0.9300
N4—C5	1.353 (3)	C18—H18	0.9300
N4—C3	1.451 (3)	C18—C17	1.388 (4)
N2—H2	0.8600	C4—H4A	0.9600
N2—C2	1.437 (3)	C4—H4B	0.9600
N2—C1	1.357 (3)	C4—H4C	0.9600
C6—C11	1.386 (3)	C12—H12A	0.9600
C6—C7	1.387 (3)	C12—H12B	0.9600
C13—C14	1.379 (3)	C12—H12C	0.9600
C13—C18	1.374 (3)	C17—H17	0.9300
C2—H2A	0.9800	C19—H19A	0.9600
C2—C3	1.542 (3)	C19—H19B	0.9600
C9—C10	1.398 (3)	C19—H19C	0.9600
			
C9—O2—C12	117.5 (2)	C10—C11—H11	120.2
C16—O1—C19	117.4 (2)	C9—C10—H10	119.9
C6—N3—C2	122.93 (17)	C11—C10—C9	120.1 (2)
C5—N3—C6	124.84 (18)	C11—C10—H10	119.9
C5—N3—C2	112.21 (17)	C9—C8—H8	120.2
C13—N1—C3	121.96 (18)	C9—C8—C7	119.6 (2)
C1—N1—C13	126.11 (18)	C7—C8—H8	120.2
C1—N1—C3	111.89 (17)	C6—C7—C8	120.3 (2)
C5—N4—H4	123.6	C6—C7—H7	119.9
C5—N4—C3	112.89 (18)	C8—C7—H7	119.9
C3—N4—H4	123.6	C13—C14—H14	119.7
C2—N2—H2	123.9	C13—C14—C15	120.6 (2)
C1—N2—H2	123.9	C15—C14—H14	119.7
C1—N2—C2	112.18 (18)	O1—C16—C15	124.9 (2)
C11—C6—N3	119.63 (19)	O1—C16—C17	115.4 (2)
C11—C6—C7	120.1 (2)	C15—C16—C17	119.7 (2)
C7—C6—N3	120.3 (2)	C14—C15—H15	120.2
N3—C5—S2	126.03 (16)	C16—C15—C14	119.7 (2)
N4—C5—S2	125.21 (16)	C16—C15—H15	120.2
N4—C5—N3	108.73 (19)	C13—C18—H18	120.1
C14—C13—N1	119.9 (2)	C13—C18—C17	119.7 (2)
C18—C13—N1	120.11 (19)	C17—C18—H18	120.1
C18—C13—C14	119.9 (2)	C3—C4—H4A	109.5
N3—C2—H2A	112.0	C3—C4—H4B	109.5
N3—C2—C3	102.70 (17)	C3—C4—H4C	109.5
N2—C2—N3	112.86 (19)	H4A—C4—H4B	109.5
N2—C2—H2A	112.0	H4A—C4—H4C	109.5
N2—C2—C3	104.61 (17)	H4B—C4—H4C	109.5
C3—C2—H2A	112.0	O2—C12—H12A	109.5
O2—C9—C10	114.6 (2)	O2—C12—H12B	109.5
O2—C9—C8	125.1 (2)	O2—C12—H12C	109.5
C8—C9—C10	120.2 (2)	H12A—C12—H12B	109.5
N1—C1—S1	126.59 (16)	H12A—C12—H12C	109.5
N1—C1—N2	109.29 (19)	H12B—C12—H12C	109.5
N2—C1—S1	124.11 (17)	C16—C17—H17	119.8
N1—C3—C2	101.56 (17)	C18—C17—C16	120.3 (2)
N1—C3—C4	111.68 (18)	C18—C17—H17	119.8
N4—C3—N1	111.96 (19)	O1—C19—H19A	109.5
N4—C3—C2	103.31 (17)	O1—C19—H19B	109.5
N4—C3—C4	112.76 (19)	O1—C19—H19C	109.5
C4—C3—C2	114.8 (2)	H19A—C19—H19B	109.5
C6—C11—H11	120.2	H19A—C19—H19C	109.5
C10—C11—C6	119.7 (2)	H19B—C19—H19C	109.5

### Hydrogen-bond geometry (Å, º)

*Cg* is the centroid of the C13–C18 ring.

**Table:**

*D*—H···*A*	*D*—H	H···*A*	*D*···*A*	*D*—H···*A*
N2—H2···S2^i^	0.86	2.62	3.265 (2)	133
N4—H4···S1^ii^	0.86	2.57	3.382 (2)	157
C7—H7···S1^iii^	0.93	2.87	3.786 (3)	170
C12—H12*B*···*Cg*^iv^	0.96	2.99	3.893 (3)	157

Symmetry codes: (i) *x*, −*y*+3/2, *z*−1/2; (ii) *x*, −*y*+3/2, *z*+1/2; (iii) −*x*+1, *y*+1/2, −*z*+1/2; (iv) *x*−1, *y*, *z*.
