# Supplementary material for: Crystal structure and Hirshfeld surface analysis of a new di­thio­glycoluril: 1,4-bis­(4-meth­oxy­phen­yl)-3a-methyl­tetra­hydro­imidazo[4,5-d]imidazole-2,5(1H,3H)-di­thione
Source: Acta Crystallogr E Crystallogr Commun. 2019 Aug 6;75(Pt 9):1297–300. doi: 10.1107/S2056989019010764 (PMC6727056; doi:10.1107/S2056989019010764)
Supplement: Supplementary file 4 [file e-75-01297-sup3.docx]

**Supplementary information**

**Crystal structure of 1,4-bis(4-methoxyphenyl)-3a-methyltetrahydroimidazo[4,5-*d*]imidazole-2,5(1*H*,3*H*)-dithione**

**Computing details**

**1,4-bis(4-methoxyphenyl)-3a-methyltetrahydroimidazo[4,5-*d*imidazole-2,5(1*H*,3*H*)-dithione**

| Crystal data |  |
| --- | --- |
| C_19_H_20_N_4_O_2_S_2_ | *F*(000) = 840 |
| M_r_ = 400.51 | D_x_ = |
| Monoclinic, P2_1_c | Mo K*α* radiation |
| a = 13.1955(3) Å | Cell parameters from 25390 reflections |
| b = 10.0157(2) Å | θ = |
| c = 14.5476(3) Å | µ= 2.725mm^-1^ |
| *α,*= 90 | T= 100.00(10) K |
| *β* = 98.392(2) ^o^ | Plate-like, colourless |
| *γ* = 90 |  |
| V= 1902.36(7) Å^3^ |  |
| *Z* = 4 |  |
|  |  |
| Data collection |  |
| Oxford Rigaku four-circle diffractometer equipped with PhotonJet (Cu, λ=1.54184 Å) X-ray Source | 3824 independent reflections |
| Absorption correction, Multi Scan; CrysAlis-Pro program package. Rigaku Oxford diffraction. | 3824 reflections with *I* > 2*σ*(*I*) |
|  | *R*_int_ = 0.0530 |
|  | *θ*_max_ = 148.398°, *θ*_min_ = 6.77° |
|  | *h* = −16→15 |
|  | *k* = −12→12 |
| *T*_min_*T*_max_0.73484- 1.00000 | *l* = −18→15 |
| 11665 measured reflections | Multi-scan |
|  |  |
| Refinement |  |
| Refinement on *F^2^* | 247 parameters |
| Least-square matrix : full | 0 restraints |
| *R* [*F^2^*>2*σ*(*F^2^*)]= 0.0470 | Primary atom site location: structure-invariant direct methods |
| *wR(F^2^)*= 0.1491 | Secondary atom site location: difference Fourier map |
| *S* = 1.222 |  |
| 25390 reflections |  |
| Hydrogen site location: inferred from neighbouring sites | ∆*ρ*_max_= 0.63 e Å^-3^ |
|  | ∆*ρ*_min_ = -0.53 Å^-3^ |

**Special Details**

**Geometry**

Fractional Atomic Coordinates (×10^4^) and Equivalent Isotropic Displacement Parameters (Å^2^×10^3^). U_eq_ is defined as 1/3 of of the trace of the orthogonalised U_IJ_ tensor.

| **Atom** | ***x*** | ***Y*** | ***Z*** |
| --- | --- | --- | --- |
| S2 | 4201.5(4) | 6375.8(5) | 5635.7(4) |
| S1 | 6930.3(4) | 6403.9(6) | 2055.0(4) |
| O2 | 523.5(12) | 5223.9(19) | 2465.6(13) |
| O1 | 10625.1(13) | 6869(2) | 5560.5(14) |
| N3 | 4296.9(13) | 7381.2(19) | 3926.2(13) |
| N1 | 6684.7(13) | 7611.1(19) | 3671.5(13) |
| N4 | 5674.4(13) | 7746(2) | 4942.8(13) |
| N2 | 5281.3(13) | 7489(2) | 2645.1(13) |
| C6 | 3331.1(15) | 6860(2) | 3521.7(14) |
| C5 | 4737.0(15) | 7169(2) | 4815.0(15) |
| C13 | 7714.4(15) | 7412(2) | 4123.2(15) |
| C2 | 4945.4(16) | 8198(2) | 3404.8(15) |
| C9 | 1427.4(16) | 5846(2) | 2795.5(16) |
| C1 | 6292.4(16) | 7179(2) | 2814.8(15) |
| C3 | 5924.4(16) | 8391(2) | 4110.9(16) |
| C11 | 3189.8(16) | 5490(2) | 3458.5(16) |
| C10 | 2237.4(17) | 4982(2) | 3102.9(17) |
| C8 | 1576.9(17) | 7205(3) | 2827.6(17) |
| C7 | 2536.0(17) | 7716(3) | 3188.2(17) |
| C14 | 8456.2(18) | 8354(3) | 4033.4(17) |
| C16 | 9685.8(17) | 7111(3) | 5058.1(18) |
| C15 | 9448.2(18) | 8197(3) | 4490.8(17) |
| C18 | 7954.4(19) | 6308(2) | 4673(2) |
| C4 | 6257.8(19) | 9834(3) | 4276.0(19) |
| C12 | -379.3(18) | 6032(3) | 2306(2) |
| C17 | 8940(2) | 6156(3) | 5143(2) |
| C19 | 11439.1(19) | 7756(4) | 5431(2) |

Anisotropic Displacement Parameters (Å^2^×10^3^). The Anisotropic displacement factor exponent takes the form: -2π^2^[h^2^a*^2^U_11_+2hka*b*U_12_+…].

| **Atom** | **U_11_** | **U_22_** | **U_33_** | **U_23_** | **U_13_** | **U_12_** |
| --- | --- | --- | --- | --- | --- | --- |
| S2 | 23.1(3) | 35.2(3) | 28.1(3) | 0.66(19) | 3.5(2) | -3.11(18) |
| S1 | 27.4(3) | 38.5(3) | 32.3(3) | -4.9(2) | 4.6(2) | 5.0(2) |
| O2 | 23.8(8) | 42.5(10) | 54.0(11) | -12.8(8) | -5.2(7) | 2.2(7) |
| O1 | 24.2(8) | 61.2(13) | 61.7(12) | -9.3(10) | -4.3(8) | 3.6(8) |
| N3 | 20.7(8) | 35.9(10) | 28.3(9) | 2.1(7) | 4.3(7) | -1.4(7) |
| N1 | 20.3(8) | 33.4(10) | 31.3(9) | -3.8(7) | 4.9(7) | 0.5(7) |
| N4 | 21.8(8) | 41.0(11) | 28.1(9) | -2.0(8) | 5.0(7) | -4.1(7) |
| N2 | 22.3(8) | 43.9(11) | 29.8(10) | -1.4(8) | 2.8(7) | 3.4(8) |
| C6 | 21.8(10) | 36.8(12) | 24.5(10) | -1.1(9) | 3.1(8) | 1.2(8) |
| C5 | 21.9(9) | 29.0(10) | 29.9(10) | -3.4(8) | 3.7(8) | 1.4(8) |
| C13 | 23.0(10) | 33.6(11) | 29.4(11) | -7.3(9) | 5.5(8) | -3.7(8) |
| C2 | 25.3(10) | 32.1(11) | 29.8(11) | 2.1(9) | 6.9(8) | 1.9(8) |
| C9 | 25.1(11) | 39.7(12) | 31.9(11) | -7.4(9) | 0.6(8) | 3.7(9) |
| C1 | 27.9(10) | 29.8(10) | 29.5(11) | 1.2(8) | 5.5(8) | -0.1(8) |
| C3 | 26.1(10) | 32.2(11) | 31.8(11) | -3.5(9) | 10.1(8) | -0.5(8) |
| C11 | 24.8(10) | 36.2(12) | 34.9(12) | -3.4(9) | -2.0(8) | 7.4(9) |
| C10 | 29.1(11) | 32.2(12) | 43.0(13) | -4.6(10) | -2.7(9) | 1.6(9) |
| C8 | 25.1(10) | 39.7(13) | 37.8(12) | 0.6(10) | 1.2(9) | 8.1(9) |
| C7 | 28.6(11) | 34.1(12) | 39.7(12) | 0.7(10) | 1.0(9) | 2.2(9) |
| C14 | 28.9(11) | 45.1(13) | 36.8(12) | 4.5(10) | 4.1(9) | -8.3(10) |
| C16 | 25.9(11) | 48.7(14) | 39.5(13) | -9.1(11) | 1.8(9) | 1.9(10) |
| C15 | 28.2(11) | 51.9(15) | 38.7(13) | -2.6(11) | 7.4(9) | -9.8(10) |
| C18 | 28.1(11) | 34.0(12) | 51.7(15) | -1.1(10) | 2.0(10) | -3.9(9) |
| C4 | 38.2(12) | 33.4(12) | 47.8(14) | -4.8(10) | 12.8(10) | -1.5(10) |
| C12 | 24.0(11) | 50.7(15) | 51.8(15) | -7.0(12) | 0.2(10) | 3.5(10) |
| C17 | 34.3(13) | 37.5(13) | 55.7(16) | 4.0(11) | -0.5(11) | 2.6(10) |
| C19 | 23.7(12) | 103(3) | 50.1(16) | -10.6(16) | 5.4(11) | -8.3(14) |

| *Hydrogen bond geometry(Å,^o^)* | | | | | |
| --- | --- | --- | --- | --- | --- |
| *D-H---A* | *D-H* | *H---A* | *D----A* | *D-H---A* | *symmetry* |
| N2-H2—S2 | 0.860 | 2.616 | 3.265 | 133.14 | x,1.5-y,1/2+z |
| N4-H4---S1 | 0.860 | 2.574 | 3.382 | 156.90 | x,1.5-y,1/2+z |

**Bond Angles.**

| **Atom** | **Atom** | **Atom** | **Angle/˚** |  | **Atom** | **Atom** | **Atom** | **Angle/˚** |
| --- | --- | --- | --- | --- | --- | --- | --- | --- |
| C9 | O2 | C12 | 117.5(2) |  | O2 | C9 | C8 | 125.1(2) |
| C16 | O1 | C19 | 117.4(2) |  | C8 | C9 | C10 | 120.2(2) |
| C6 | N3 | C2 | 122.93(17) |  | N1 | C1 | S1 | 126.59(16) |
| C5 | N3 | C6 | 124.84(18) |  | N1 | C1 | N2 | 109.29(19) |
| C5 | N3 | C2 | 112.21(17) |  | N2 | C1 | S1 | 124.11(17) |
| C13 | N1 | C3 | 121.96(18) |  | N1 | C3 | C2 | 101.56(17) |
| C1 | N1 | C13 | 126.11(18) |  | N1 | C3 | C4 | 111.68(18) |
| C1 | N1 | C3 | 111.89(17) |  | N4 | C3 | N1 | 111.96(19) |
| C5 | N4 | C3 | 112.89(18) |  | N4 | C3 | C2 | 103.31(17) |
| C1 | N2 | C2 | 112.18(18) |  | N4 | C3 | C4 | 112.76(19) |
| C11 | C6 | N3 | 119.63(19) |  | C4 | C3 | C2 | 114.8(2) |
| C11 | C6 | C7 | 120.1(2) |  | C10 | C11 | C6 | 119.7(2) |
| C7 | C6 | N3 | 120.3(2) |  | C11 | C10 | C9 | 120.1(2) |
| N3 | C5 | S2 | 126.03(16) |  | C9 | C8 | C7 | 119.6(2) |
| N4 | C5 | S2 | 125.21(16) |  | C6 | C7 | C8 | 120.3(2) |
| N4 | C5 | N3 | 108.73(19) |  | C13 | C14 | C15 | 120.6(2) |
| C14 | C13 | N1 | 119.9(2) |  | O1 | C16 | C15 | 124.9(2) |
| C18 | C13 | N1 | 120.11(19) |  | O1 | C16 | C17 | 115.4(2) |
| C18 | C13 | C14 | 119.9(2) |  | C15 | C16 | C17 | 119.7(2) |
| N3 | C2 | C3 | 102.70(17) |  | C16 | C15 | C14 | 119.7(2) |
| N2 | C2 | N3 | 112.86(19) |  | C13 | C18 | C17 | 119.7(2) |
| N2 | C2 | C3 | 104.61(17) |  | C18 | C17 | C16 | 120.3(2) |
| O2 | C9 | C10 | 114.6(2) |  |  |  |  |  |

**Bond Lengths**

| **Atom** | **Atom** | **Length/Å** | **Atom** | **Atom** | **Length/Å** |
| --- | --- | --- | --- | --- | --- |
| S2 | C5 | 1.674(2) | N2 | C1 | 1.357(3) |
| S1 | C1 | 1.674(2) | C6 | C11 | 1.386(3) |
| O2 | C9 | 1.370(3) | C6 | C7 | 1.387(3) |
| O2 | C12 | 1.431(3) | C13 | C14 | 1.379(3) |
| O1 | C16 | 1.366(3) | C13 | C18 | 1.374(3) |
| O1 | C19 | 1.427(4) | C2 | C3 | 1.542(3) |
| N3 | C6 | 1.423(3) | C9 | C10 | 1.398(3) |
| N3 | C5 | 1.355(3) | C9 | C8 | 1.375(4) |
| N3 | C2 | 1.472(3) | C3 | C4 | 1.520(3) |
| N1 | C13 | 1.435(3) | C11 | C10 | 1.385(3) |
| N1 | C1 | 1.350(3) | C8 | C7 | 1.395(3) |
| N1 | C3 | 1.487(3) | C14 | C15 | 1.389(3) |
| N4 | C5 | 1.353(3) | C16 | C15 | 1.374(4) |
| N4 | C3 | 1.451(3) | C16 | C17 | 1.391(4) |
| N2 | C2 | 1.437(3) | C18 | C17 | 1.388(4) |
